# Characterization of Ethos therapy systems for adaptive radiation therapy: A multi‐machine comparison

**DOI:** 10.1002/acm2.13905

**Published:** 2023-01-17

**Authors:** Agustinus J. van de Schoot, Daan Hoffmans, Karel M. van Ingen, Martijn J. Simons, Jan Wiersma

**Affiliations:** ^1^ Department of Radiation Oncology Amsterdam University Medical Center – location University of Amsterdam Amsterdam the Netherlands; ^2^ Department of Radiation Oncology Amsterdam University Medical Center – location Vrije Universiteit Amsterdam Amsterdam the Netherlands

**Keywords:** adaptive radiotherapy, beam characterization, machine commissioning, quality assurance

## Abstract

**Purpose:**

The recently released Ethos therapy system (Varian Medical Systems) allows for online CBCT‐guided adaptive radiation therapy (RT). The clinical introduction of multiple systems requires machine characterization and machine variation quantification to allow patient interchangeability between systems. Despite several clinical introductions, limited vendor‐independent information on machine performance is available. Our aim was to determine the relevant dosimetric and mechanical characteristics of individual machines and to quantify machine variations.

**Methods:**

Six Ethos treatment machines, equipped with a 6‐MV FFF beam including dual‐layer MLC and kV‐CBCT system, were recently introduced clinically after extensive machine characterization and pre‐configured beam model verification. Point doses and profiles were measured and compared to vendor‐provided reference data and dose calculations. Also, dose calculations were verified based on point measurements for non‐standard fields and dose distributions for optimized treatment plans. Agreements between dose profiles (dose distributions) were quantified using 1D (3D) γ‐analysis. Additionally, we quantified leaf transmission, dosimetric leaf gap (DLG) and couch attenuation, determined isocenter accuracy and kV‐MV isocenter coincidence and verified the kV‐CBCT system. Machine variations were quantified for all dosimetric and mechanical characteristics.

**Results:**

For all machines, distinct agreements were found between measurements and vendor‐provided reference data as well as measurements and dose calculations. Mean γ_1%/1mm_ values for all profiles were below 0.30. All profiles, point measurements and dose distributions matched well among the six machines. Minimal machine variations were found in terms of DLG (0.05 mm), leaf transmission (0.001%), isocenter accuracy (0.08 mm), kV‐MV isocenter coincidence (0.15 mm), couch attenuation (0.69%), and CBCT imaging dose (0.29 mGy).

**Conclusions:**

This study demonstrates excellent agreement between individual Ethos therapy systems and vendor‐provided reference data as well as a pre‐configured beam model. Furthermore, our results show good consistency among all machines and provide valuable insights on relevant machine characteristics. The systematically obtained results provide benchmark data for future clinical introduction of Ethos therapy systems.

## 1 INTRODUCTION

Radiation therapy (RT) aims to deliver the prescribed dose to the target while minimizing dose to healthy surrounding tissue. The clinical application of external beam RT along with beam modulation enables highly conformal dose distributions including steep dose gradients to minimize healthy tissue irradiation. Interfraction anatomical changes however limit the efficacy of the advanced treatment technique, resulting in the use of substantial safety margins. Adaptive RT (ART) has the potential to enhance treatment precision by adapting the radiation delivery to the anatomical changes based on pre‐fraction imaging and consequently reducing safety margins.^1^, ^2^ Although magnetic resonance imaging provides outstanding soft tissue visualization and functional imaging possibilities for advanced adaptive strategies,^3^, ^4^, ^5^ the integrated cone‐beam computed tomography (CBCT) imaging system on conventional treatment machines is widely used for treatment adaptation purposes. Different CBCT‐guided adaptive strategies for various anatomical sites have been introduced,^6^, ^7^, ^8^, ^9^ however treatment adaptation including online contouring and plan optimization based on pre‐fraction CBCT imaging remains challenging.

The recently released Ethos therapy system (Varian Medical Systems) allows for CBCT‐guided online ART using artificial intelligence‐driven automatic contouring and plan optimization.^10^ The system consists of a ring‐mounted linear accelerator equipped with a kV‐CBCT imaging device, a dedicated treatment planning system (TPS) with a pre‐configured beam model, an adaptive treatment platform to enable online adaptive workflows and integrated machine performance checks to facilitate daily quality assurance. Although the system enables online plan adaptations for treatment precision enhancement, the treatment accuracy is largely affected by the quality of the system. Clinical introduction of online ART therefore requires extensive system commissioning including machine characterization and TPS validation.

The Ethos therapy system has been adopted in different clinics for patient treatments including online plan adaptations.^11^, ^12^ Nevertheless, limited vendor‐independent information related to machine performances is available. Several studies reported on machine commissioning and beam model validation of the Ethos therapy system,^13^, ^14^, ^15^ however none of them performed an extensive and systematical characterization and comparison of multiple Ethos therapy systems. Machine commissioning studies were solely based on the previous generation linear accelerator and the performances of machines integrated within the Ethos therapy platform are unknown. Moreover, none of them reported on the variability in machine characteristics while minimal machine variation is a prerequisite for machine matching to allow for patient interchangeability between treatment units in both adaptive and non‐adaptive treatments.

To investigate the characteristics of an individual Ethos therapy system as well as the variation in characteristics between Ethos therapy systems, performances of all relevant treatment machine components need to be determined based on extensive and systematical commissioning of multiple treatment machines. Therefore, the aim of this study was to determine the dosimetric and mechanical characteristics of relevant treatment machine components for individual Ethos therapy systems and to quantify the variability in machine characteristics between Ethos therapy systems.

## 2 MATERIAL AND METHODS

Between May 2020 and January 2022 six Ethos therapy systems were installed consecutively in our department. After machine acceptance, the clinical introduction of individual systems required extensive and systematical machine commissioning consisting of beam characterization, geometric accuracy quantification, kV‐CBCT system validation and couch attenuation verification. All commissioning components were based on published practical guidelines.^16^, ^17^, ^18^, ^19^, ^20^, ^21^

### 2.1 Beam characteristics

Characterization of the 6‐MV flattening filter free (FFF) beam consisted of beam shaping device validation, beam quality determination, and dosimetric verification including beam model verification. Since the pre‐configured beam model has been built by the vendor based on their measured reference data (Varian representative beam data), measured dosimetric characteristics were first compared to the vendor‐provided reference data. Next, the agreement between machines and pre‐configured beam model was determined by comparing measurements and calculations for both symmetrical open fields and optimized treatment plans.

#### 2.1.1 Beam shaping device

Ethos machines utilizes a dual‐layer multileaf collimator (MLC) design. The proximal (distal) MLC leaf bank consists of 29 (28) leaf pairs and the banks are positioned half leaf apart to introduce an effective leaf width of 5.0 mm while minimizing interleaf leakage. MLC characterization consisted of dosimetric leaf gap (DLG) determination and leaf transmission verification for both individual and combined leaf banks. The DLG represents the modelled effect of the rounded MLC leaf ends and was derived by the interception of the leaf gap axis after linear extrapolation of the obtained relation between leaf gaps and measured output.^22^

Leaf transmission for individual (combined) leaf banks was defined as the fraction of output measured for one (both) closed leaf bank and the 26 × 26 cm^2^ open field, according to the standard procedure of the vendor. All measurements were obtained with an ionization chamber (IC) at isocenter position perpendicular to leaf travel direction inside a solid water phantom.

#### 2.1.2 Beam quality

The quality of the beam, indicated by the quality index (QI), was quantified by the ratio of ionizations measured (PTW Semiflex3D or IBA CC13) on the central axis at depths of 20.0 cm and 10.0 cm in water using a 10 × 10 cm^2^ open field and a constant source‐detector distance of 100.0 cm.

#### 2.1.3 Initial beam verification

Dosimetric verification for symmetrical open fields ranging from 2 × 2 cm^2^ till 28 × 28 cm^2^ included point dose measurements as well as the acquisition of percentage depth dose (PDD) profiles and crossline profiles at different depths. For the largest field, also diagonal profiles were collected at different depths. All measurements were executed in accordance with the vendor‐provided reference measurements to minimize deviations induced by set‐up discrepancies, resulting in the use of either PTW Beamscan water tank combined with PTW Semiflex3D detector or IBA BluePhantom II water tank combined with IBA CC13 detector. Point dose measurements for output factor determination were obtained using a source‐surface distance (SSD) of 95.0 cm with the effective point of measurement of the IC positioned in the isocenter 5.0 cm below the water surface. For all measurements, accurate positioning of measurement equipment relative to the beam isocenter was ensured by verification of the SSD and IC position using MV imaging projections.

After resampling all profile measurements to a 1.0 mm resolution, PDD profiles were normalized at the pre‐defined dose maximum position of 13.0 mm while for crossline profiles the profile point at the central axis was used. The agreement between measured and vendor‐provided reference profiles was quantified using the 1D γ‐analysis (1%/1 mm). For PDD profiles also the relative dose at 10.0 cm depth (D_10cm_) was extracted. Field sizes for crossline profiles were derived by calculating the full width at half maximum (FWHM) based on the inflection point of a polynomial fit in penumbra regions and compared to nominal field sizes.^23^, ^24^ Penumbra regions were defined between positions where the measured dose equals 0.4 respectively 1.6 times the inflection point dose. A γ_1%/1mm_ evaluation was performed for the profile bounded by the penumbra regions as well as the penumbra regions alone. Variations between machines were derived by calculating average and standard deviation (SD) values of all machine results. All data processing and analyses were performed in MATLAB (The MathWorks Inc.). Output factors were calculated by the ratio between point doses and the point dose for the 10 × 10 cm^2^ reference field and differences between vendor‐provided and derived output factors were quantified.

#### 2.1.4 Beam model verification

The agreement between machines and beam model was verified by quantifying differences between measurements and calculations for static dose delivery using open fields and dynamic dose delivery using treatment plans. After position verification of measurement equipment using MV imaging projections, open field measurements were collected in a water tank (PTW Beamscan, IBA BluePhantom II) using fields ranging from 1 × 1 cm^2^ till 28 × 28 cm^2^ and included the acquisition of PDD profiles over the central axis, crossline and inline profiles at different depths for symmetric and off‐axis configurations and point dose measurements.

PDD profiles for large open fields (>5 × 5 cm^2^) were acquired using a cylindrical IC (PTW Semiflex3D, IBA CC13) while all other profile measurements were performed with a PTW microDiamant detector. The agreement between measured and calculated profiles was determined by extracting γ_1%/1mm_ results for all profiles, D_10cm_ values for PDD profiles and FWHM‐based field sizes for crossline and inline profiles.

Point measurements for squared and elongated fields were performed using the PTW microDiamant detector for small fields (≤5 × 5 cm^2^) and a cylindrical IC (PTW Semiflex3D or IBA CC13) for larger fields (≥5 × 5 cm^2^) at 10.0 cm depth with a SSD of 90.0 cm. Output factors, the ratio between point doses and the point dose for the reference 10 × 10 cm^2^ field, were calculated using a daisy‐chaining method for small fields^25^ and compared to output factors based on point dose calculations.

Additionally, beam model verification for non‐standard circumstances was assessed by calculating differences between measured and calculated point doses for non‐standard field configurations at different depths and off‐axis fields with non‐standard SSD values (Table 1). All point measurements for non‐standard field configurations were converted to point doses using measurement results for reference circumstances collected at the same time.

**TABLE 1:** Overview of non‐standard field configurations and measurement positions used for point dose measurements.

	Field configuration				Measurement position	
				CAX offset [cm]	CAX offset [cm]	
	Field size [cm^2^]	Collimator [°]	SSD [cm]	Inline; crossline	Inline; crossline	Depth [cm]
**NORM**	10.0 × 10.0	90.0	90.0	0.0; 0.0	0.0; 0.0	10.0
**I**	10.0 × 10.0	90.0	93.0	0.0; 0.0	0.0; 0.0	5.0
	10.0 × 10.0	90.0	93.0	0.0; 0.0	0.0; 0.0	7.0
	10.0 × 10.0	90.0	93.0	0.0; 0.0	0.0; 0.0	15.5
	10.0 × 10.0	90.0	93.0	0.0; 0.0	0.0; 0.0	24.8
**II**	4.5 × 4.5	90.0	93.0	0.0; 0.0	0.0; 0.0	5.0
**III**	4.5 × 4.5	90.0	93.0	10.0; 0.0	9.8; 0.0	5.0
	4.5 × 4.5	90.0	93.0	10.0; 0.0	10.8; 0.0	15.0
**IV**	4.5 × 4.5	0.0	93.0	0.0; 10.0	0.0; 9.8	5.0
	4.5 × 4.5	0.0	93.0	0.0; 10.0	0.0; 10.8	15.0
**V**	17.0 × 23.0	90.0	93.0	0.0; 0.0	0.0; 0.0	5.0
**VI**	7.5 × 7.5	90.0	93.0	10.0; 0.0	9.8; 0.0	5.0
	7.5 × 7.5	90.0	93.0	10.0; 0.0	10.3; 0.0	10.0
**VII**	10.0 × 10.0	90.0	93.0	5.0; 0.0	5.1; 0.0	8.0
**VIII**	*MLC shaped*	90.0	93.0	0.0; 0.0	0.0; 0.0	8.0
**IX**	*MLC shaped*	90.0	93.0	0.0; 0.0	0.0; 0.0	8.0
**X**	*MLC shaped*	90.0	93.0	0.0; 0.0	0.0; 0.0	8.0
**XI**	10.0 × 10.0	90.0	82.0	0.0; 0.0	0.0; 0.0	5.0
	10.0 × 10.0	90.0	82.0	0.0; 0.0	0.0; 0.0	14.8
	10.0 × 10.0	90.0	82.0	0.0; 0.0	0.0; 0.0	18.0
	10.0 × 10.0	90.0	82.0	0.0; 0.0	0.0; 0.0	28.6
**XII**	3.5 × 3.5	0.0	82.0	0.0; 0.0	0.0; 0.0	5.5
**XIII**	3.5 × 3.5	33.0	82.0	0.0; 0.0	0.0; 0.0	5.5
**XIV**	18.5 × 18.5	90.0	82.0	0.0; 0.0	0.0; 0.0	27.0
**XV**	3.5 × 3.5	90.0	82.0	0.0; 0.0	0.0; 0.0	27.0
**XVI**	3.5 × 3.5	90.0	82.0	12.4; 0.0	11.0; 0.0	7.0
**XVII**	13.0 × 13.0	90.0	82.0	6.5; 0.0	5.8; 0.0	7.0
	13.0 × 13.0	90.0	82.0	6.5; 0.0	6.9; 0.0	24.0
**XVIII**	13.0 × 13.0	90.0	82.0	6.5; 6.5	5.9; 5.9	9.0
**XIX**	*MLC shaped*	0.0	82.0	0.0; 0.0	0.0; 0.0	8.0
**XX**	*MLC shaped*	0.0	82.0	0.0; 0.0	0.0; 0.0	8.0
**XXI**	5.0 × 5.0	0.0	105.0	8.4; 2.5	9.0; 2.7	7.0

Abbreviations: CAX, central axis; MLC, multileaf collimator; SSD, source‐surface distance; NORM, normalization field.

For various tumor sites, dose distributions of optimized intensity‐modulated radiation therapy (IMRT) and volumetric‐modulated arc therapy (VMAT) treatment plans were measured using the PTW Octavius 4D phantom and corrected for daily machine output variations. The agreement between measured and calculated dose distributions was quantified using 3D γ_3%/3mm_ analyses and evaluated based on pass rates for low (10%, 30%), intermediate (50%), and high (80%, 90%) dose levels.^18^ Per dose level (threshold), voxels with a dose value of at least the threshold percentage of the maximum dose were included. The variation between machines was assessed by comparing measured dose distributions using 3D γ_1%/1mm_ evaluations after 3D alignment corrections. Given the strict evaluation criteria, average alignment corrections were calculated on a per phantom position basis to minimize phantom position errors. All 3D alignments and evaluations were performed using the PTW VeriSoft software (version 7.2).

### 2.2 Machine characteristics

#### 2.2.1 Geometry

Geometric verification consisted of isocenter accuracy determination for both gantry and collimator rotations and isocenter coincidence quantification of the MV beam and kV‐CBCT system. Isocenter accuracy was determined based on spoke‐shot analysis after irradiation of radiographic film (Gafchromic EBT3, Ahsland) inserted in a solid water phantom. For gantry rotation analysis, spoke‐shots for clockwise (CW) and counter‐clockwise (CCW) gantry rotations were acquired with radiographic film in the axial plane. Spoke‐shots for collimator rotation analysis were obtained using a gantry position of 0° (90°) with radiographic film in the coronal (sagittal) orientation. Irradiated films were scanned using a flatbed scanner (Epson Perfection V700) and analyzed using an in‐house developed tool.

The kV‐MV isocenter alignment was quantified using the MIMI^TM^ phantom (Standard Imaging, Inc.). After manual alignment based on machine lasers, the obtained 3D kV‐CBCT image of the phantom was registered to the initially acquired CT of the phantom. Couch position corrections resulting from image registration were applied to align the center of the phantom to the kV‐CBCT isocenter. Next, two orthogonal 2D MV images were acquired and registered to the initial image. The resulting couch position corrections indicated kV‐MV isocenter discrepancies.

#### 2.2.2 kV‐CBCT system

The kV‐CBCT system was characterized by verifying beam energies, exposure times and half‐value layer (HVL) values as well as conducting CBCT dosimetry. Energy peaks and exposure times were measured based on projectional radiography and compared with intended beam energies and exposure times. Also, exposure time linearity was derived by calculating Pearson correlation coefficients for the relation between exposure times and dose. The kV‐CBCT beam quality was verified by comparing specified and measured HVL values for different energies.

CBCT dosimetry was conducted by quantifying the imaging dose using a PTW Farmer chamber positioned in a computed tomography dose index (CTDI) phantom. Imaging dose per clinical CBCT protocol was represented by the CTDI_weighted_ value, the sum of one‐third of the measured dose in the central hole of the phantom and two‐third of the average dose measured in the four peripheral holes of the phantom. The deviations between measured and vendor‐specified CTDI_weighted_ values were calculated.

#### 2.2.3 Treatment couch

Beam attenuation by the treatment couch was assessed by measuring point doses at two different couch positions because of the longitudinal couch thickness variation. Measurements were performed using a PTW Semiflex3D detector placed horizontally in the center of a cubic solid water phantom. The phantom was positioned with the center of the detector aligned with the beam isocenter and parallel to the couch longitudinal axis. Per position, measurements for three open fields (5 × 5 cm^2^, 10 × 10 cm^2^, 20 × 20 cm^2^) were performed using perpendicular (gantry positions: 0° and 180°) and oblique (gantry positions: 45° and 225°) beams. Attenuation was defined as the average percentage output difference of all field sizes measured for perpendicular beams (G_0_/G_180_) as well as oblique beams (G_45_/G_225_).

## 3 RESULTS

### 3.1 Beam characteristics

#### 3.1.1 Beam shaping device

The average DLG and leaf transmission for the combined leaf banks was –0.48 mm and 0.006% (Table 2). For all machines, the leaf transmission for combined leaf banks was below the vendor‐specified 0.01%. Similar machine performance in terms of DLG and leaf transmission was found, indicated by a DLG (leaf transmission) variability of 0.05 mm (0.001%). Individual machine results can be found in Table S1.

**TABLE 2:** Average [min–max] and standard deviation beam shaping device characterization results of all Ethos treatment machines. Results of individual machines can be found in Table S1

Multileaf collimator		Average [min–max]	SD
Leaf transmission [%]	Proximal leaf bank	0.421 [0.417–0.430]	0.004
	Distal leaf bank	0.421 [0.406–0.440]	0.013
	Combined leaf banks	0.006 [0.004–0.008]	0.001
DLG [mm]	Proximal leaf bank	0.44 [0.37–0.52]	0.06
	Distal leaf bank	0.30 [0.26–0.34]	0.03
	Combined leaf banks	−0.48 [−0.55–0.42]	0.05

Abbreviations: SD, standard deviation; DLG, dosimetric leaf gap; mm, millimeter.

#### 3.1.2 Beam quality

A similar beam penetrating ability was found for all six machines, indicated by a mean (min–max) and SD QI of 0.625 (0.624–0.628) and 0.001.

#### 3.1.3 Initial beam verification

**FIGURE 1:**
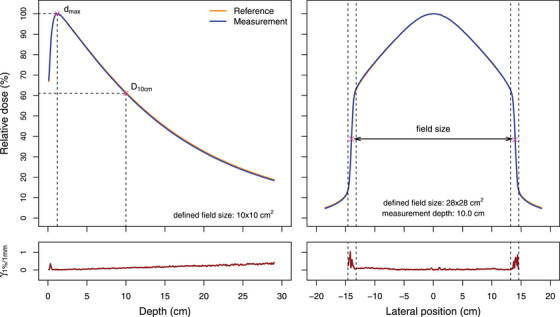
Representative example of PDD profile comparison between measured and vendor‐provided reference 10 × 10 cm^2^ fields (left) and crossline profile comparison between measured and vendor‐provided reference 28 × 28 cm^2^ fields at 10.0 cm measurement depth (right), including calculated γ_1%/1 mm_ results. The PDD profile example also indicates the position of d_max_ and D_10cm_ points while the crossline profile example shows the inflection points used for FWHM‐based field size calculation within the penumbra region indicated by vertical dotted lines.

**FIGURE 2:**
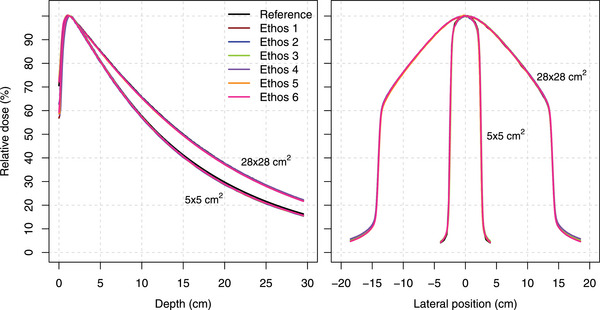
Examples of measured PDD profiles (left) and crossline profiles at 10.0 cm depth (right) for both 5 × 5 cm^2^ and 28 × 28 cm^2^ fields on all six machines including vendor‐provided reference profiles.

**FIGURE 3:**
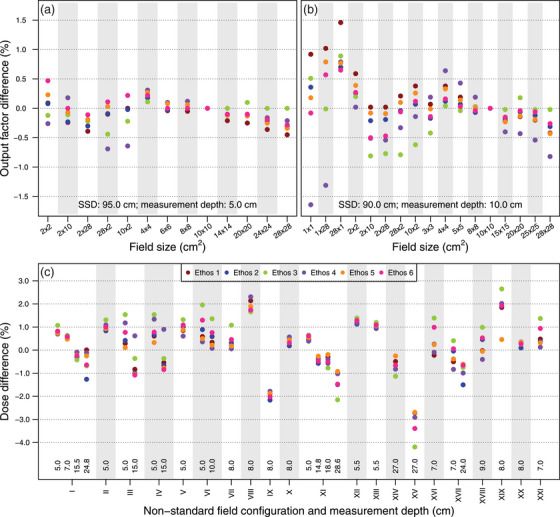
Output factor results for all machines obtained during machine characterization (a) and beam model verification (b) as well as point dose measurement results obtained during beam model verification using non‐standard field configurations (c). The Roman numerals correspond to the non‐standard field configurations defined in Table 1.

Typical examples of a PDD and crossline profile evaluation including profile parameters (Figure 1) and measured profiles for all machines are shown (Figure 2). Table 3 summarizes the D_10cm_ and FWHM‐based field size results for all machines. For all machines, the largest D_10cm_ deviation from the reference value was 0.64%, 0.44%, 0.60%, and 0.62% for the 2 × 2 cm^2^, 6 × 6 cm^2^, 10 × 10 cm^2^, and 28 × 28 cm^2^ field, respectively. The largest variation in D_10cm_ between machines was 0.27% for the 10 × 10 cm^2^ field. For crossline profiles at the main axis, the largest field size deviation from reference data of 1.09 mm was obtained for the 28 × 28 cm^2^ field. Table S2 shows all D_10cm_ and FWHM‐based field size results for individual machines. Compared to vendor‐provided reference measurements, the maximum γ_1%/1mm_ value for PDD profiles was 0.30 and all γ_1%/1mm_ results for the overall profile (penumbra region) were below 0.28 (0.89) (Table S3). Figure 3a shows minimal differences between reference and measured output factors for all machines as well as limited machine variability.

**TABLE 3:** Average [min–max] and standard deviation initial beam verification results of all Ethos treatment machines. The first section shows D_10cm_ results for PDD profiles of different fields and the second section shows field size results for crossline profiles of fields at different depths. Reference values derived from the Varian representative beam data are also shown. Results of individual machines can be found in Table S2

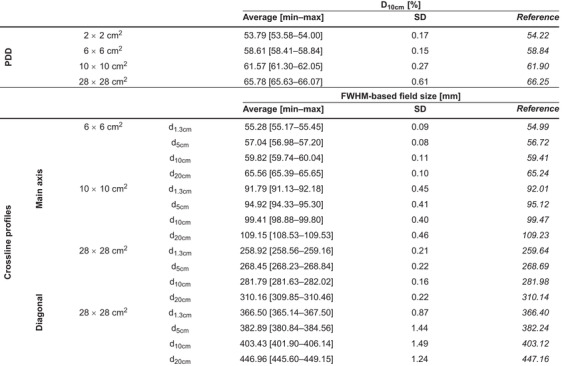

Abbreviations: SD, standard deviation; PDD, percentage depth dose; FWHM, full width at half maximum; cm, centimeter; mm, millimeter; D, dose.

#### 3.1.4 Beam model verification

The agreement between measured and calculated profiles of on‐axis open fields is shown in terms of D_10cm_ and FWHM‐based field sizes (Table 4, Table S4) as well as γ_1%/1mm_ (Table S5). The largest γ_1%/1mm_ value for PDD and crossline (inline) profiles of 0.82 and 0.91 (0.89) were all found for the 1 × 1 cm^2^ field. The largest D_10cm_ (field size) deviation of 2.29% (1.18 mm) was observed for the 1 × 1 cm^2^ (28 × 28 cm^2^) field. The maximum D_10cm_ (field size) variability of 0.83% (0.40 mm) indicated accurate machine agreement. For off‐axis open fields, all mean γ_1%/1mm_ were below 0.40 (Table S5). Output factor verification resulted in machine variations within 1.0% for squared fields (Figure 3b). For non‐standard fields, the largest dose differences between calculations and measurements were found for MLC‐shaped irregular fields or large measurement depths while the variation in point doses measured at different machines was limited (Figure 3c).

**TABLE 4:** Average [min–max] and standard deviation beam model verification results of all Ethos treatment machines. The first section shows D_10cm_ results for PDD profiles of different fields and the second section shows field size results for both crossline and inline profiles of fields at different depths. Reference values derived from Ethos TPS calculations are also shown. Results of individual machines can be found in Table S4

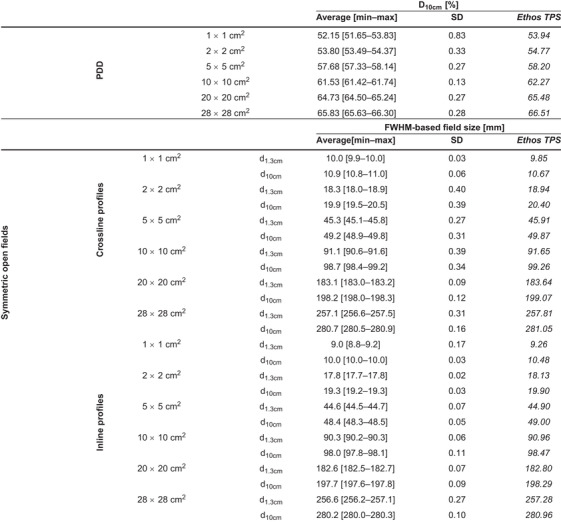

Abbreviations: SD, standard deviation; PDD, percentage depth dose; FWHM, full width at half maximum; cm, centimeter; mm, millimeter; D, dose; TPS, treatment planning system.

Table 5 shows the agreement between measured and calculated dose distributions in terms of pass rates (γ_3%/3mm_ ≤ 1.0) per dose level (threshold) for all machines. Mean pass rates for low and intermediate dose levels were all above 92.0%. For higher dose levels, the mean pass rate decreased below 92.0% for two plans. Table S6 shows the agreement between measured and calculated dose distributions for individual machines. The agreement between measured dose distributions in terms of pass rates (γ_1%/1mm_ ≤ 1.0) is shown in Table 6. For all plans, mean pass rates above 93.0% were found for low and intermediate dose levels. One plan showed mean pass rate below 93.0% for high dose levels. Due to strict evaluation criteria, substantial pass rate variations were found in few plans for the higher dose levels as well as the low and intermediate dose levels (Table 6, Table S7).

**TABLE 5:** Average [min–max] and standard deviation gamma pass rates per dose level of all Ethos treatment machines. Pass rate values (γ_3%/3mm (local dose)_ ≤ 1.0) per dose level represent the agreement between measured dose distributions and calculated dose distributions using the Ethos TPS. Results of individual machines can be found in Table S6

Plan ID	Tumor site	Treatment technique		Fractionation	Average [min–max]; SD				
					10%	30%	50%	80%	90%
1	Brain	IMRT	12 fields	5 × 4.0 Gy	98.2 [97.4–98.6]; 0.4	98.7 [98.4–99.1]; 0.3	98.8 [98.3–99.1]; 0.3	99.0 [98.3–99.4]; 0.4	99.7 [99.6–99.9]; 0.1
2	Brain	VMAT	3 arcs	5 × 4.0 Gy	98.6 [97.4–99.0]; 0.6	98.9 [98.3–99.3]; 0.4	99.1 [98.8–99.5]; 0.3	98.9 [98.5–99.4]; 0.3	99.8 [99.7–99.9]; 0.1
3	Breast	IMRT	4 fields	5 × 5.7 Gy	97.7 [96.6–98.1]; 0.6	97.7 [96.8–98.3]; 0.6	97.4 [96.3–98.1]; 0.7	91.4 [85.7–94.5]; 3.4	58.2 [30.5–75.4]; 16.0
4	Breast	VMAT	3 arcs	5 × 5.2 Gy	95.8 [94.6–96.8]; 0.7	97.2 [96.4–98.2]; 0.7	97.2 [96.4–98.2]; 0.6	98.0 [97.3–98.9]; 0.6	100.0 [100.0–100.0]; 0.0
5	Prostate	IMRT	9 fields	20 × 3.0 Gy	95.3 [94.8–95.9]; 0.4	96.4 [95.7–97.2]; 0.5	97.2 [95.3–98.0]; 1.0	93.8 [89.3–95.8]; 2.4	92.2 [86.4–94.7]; 3.1
6	Prostate	VMAT	3 arcs	20 × 3.0 Gy	96.8 [96.1–97.9]; 0.7	98.8 [98.4–99.3]; 0.4	98.4 [97.7–99.0]; 0.5	96.3 [94.7–97.8]; 1.2	96.1 [93.7–97.8]; 1.5
7	Prostate	VMAT	3 arcs	20 × 3.0 Gy	92.5 [90.9–95.2]; 1.7	95.7 [94.4–97.3]; 1.1	93.8 [91.7–95.7]; 1.5	85.9 [81.2–90.2]; 3.4	82.0 [75.3–87.6]; 4.6
8	Prostate	IMRT	12 fields	20 × 3.2 Gy	97.0 [96.6–97.2]; 0.2	98.7 [98.5–98.8]; 0.1	98.9 [98.4–99.3]; 0.3	97.3 [96.2–98.3]; 0.8	99.1 [98.1–99.4]; 0.5
9	Prostate	VMAT	3 arcs	20 × 3.2 Gy	96.4 [93.1–97.4]; 1.7	99.0 [98.1–99.3]; 0.4	98.6 [97.3–99.2]; 0.7	97.1 [95.0–98.2]; 1.2	99.8 [99.4–100.0]; 0.2
10	Rectum	VMAT	3 arcs	5 × 5.0 Gy	96.6 [95.1–97.6]; 0.9	97.7 [97.3–98.4]; 0.4	98.4 [97.9–99.0]; 0.4	96.9 [96.2–98.1]; 0.8	97.3 [96.2–98.3]; 0.9
11	Rectum	VMAT	3 arcs	5 × 5.0 Gy	96.5 [94.8–97.9]; 1.1	97.8 [97.2–98.7]; 0.5	98.0 [97.4–98.9]; 0.5	96.3 [95.3–97.9]; 1.0	96.9 [95.3–98.4]; 1.2

Abbreviations: SD, standard deviation; IMRT, intensity modulated radiation therapy; VMAT, volumetric modulated arc therapy; Gy, Gray; TPS, treatment planning system.

**TABLE 6:** Average [min–max] and standard deviation gamma pass rate results per dose level between Ethos treatment machines. Pass rate values (γ_1%/1mm (local dose)_ ≤ 1.0) per dose level represent the agreement between dose distributions measured on treatment machine Ethos 1 and dose distributions measured on all other Ethos treatment machines. Substantial pass rate variations are shown for plans 3 and 4. Results of individual agreements of dose distributions can be found in Table S7

Plan ID	Tumor site	Treatment technique		Fractionation	Average [min–max]; SD				
					10%	30%	50%	80%	90%
1	Brain	IMRT	12 fields	5 × 4.0 Gy	97.2 [93.5–99.6]; 2.3	96.6 [91.8–99.5]; 2.9	96.2 [90.1–99.5]; 3.6	96.2 [86.9–99.8]; 5.3	95.7 [83.9–99.8]; 6.7
2	Brain	VMAT	3 arcs	5 × 4.0 Gy	99.3 [98.0–99.9]; 0.8	99.2 [97.6–99.9]; 1.0	99.3 [97.1–99.9]; 1.2	98.8 [95.2–99.9]; 2.0	98.5 [93.8–99.8]; 2.6
3	Breast	IMRT	4 fields	5 × 5.7 Gy	96.2 [87.5–99.0]; 5.0	96.5 [89.5–98.8]; 4.0	97.0 [92.0–98.9]; 2.9	91.2 [78.7–99.6]; 8.4	78.4 [42.3–100.0]; 22.4
4	Breast	VMAT	3 arcs	5 × 5.2 Gy	93.8 [73.4–99.9]; 11.4	93.4 [73.4–99.9]; 11.2	93.7 [76.0–99.9]; 10.0	93.9 [82.8–99.9]; 7.2	95.9 [87.7–99.9]; 5.0
5	Prostate	IMRT	9 fields	20 × 3.0 Gy	95.1 [88.7–98.8]; 4.4	96.0 [89.4–99.4]; 4.4	98.4 [92.8–99.9]; 3.2	97.1 [86.3–100.0]; 6.0	96.0 [81.0–100.0]; 8.4
6	Prostate	VMAT	3 arcs	20 × 3.0 Gy	99.5 [98.5–99.9]; 0.6	99.4 [97.8–100.0]; 0.9	98.5 [94.3–100.0]; 2.4	96.6 [86.7–100.0]; 5.6	95.1 [80.8–100.0]; 8.1
7	Prostate	VMAT	3 arcs	20 × 3.0 Gy	99.2 [97.6–99.9]; 0.9	99.2 [96.8–100.0]; 1.3	98.4 [93.8–100.0]; 2.6	96.4 [86.2–100.0]; 5.9	95.0 [80.9–100.0]; 8.1
8	Prostate	IMRT	12 fields	20 × 3.2 Gy	99.0 [98.5–99.5]; 0.4	99.6 [98.9–99.9]; 0.4	99.4 [97.1–100.0]; 1.3	98.5 [92.6–100.0]; 3.3	97.4 [87.0–100.0]; 5.8
9	Prostate	VMAT	3 arcs	20 × 3.2 Gy	99.2 [98.0–99.9]; 0.7	99.6 [98.0–100.0]; 0.9	99.4 [97.1–100.0]; 1.3	99.2 [96.8–100.0]; 1.4	99.5 [98.4–100.0]; 0.7
10	Rectum	VMAT	3 arcs	5 × 5.0 Gy	99.3 [98.6–99.9]; 0.5	99.4 [98.4–99.9]; 0.6	99.8 [99.4–99.9]; 0.2	99.7 [99.3–99.9]; 0.3	99.5 [98.7–99.8]; 0.5
11	Rectum	VMAT	3 arcs	5 × 5.0 Gy	99.4 [99.0–99.9]; 0.4	99.4 [99.0–99.9]; 0.4	99.8 [99.7–99.9]; 0.1	99.7 [99.5–99.8]; 0.1	99.6 [99.4–99.8]; 0.2

Abbreviations: SD, standard deviation; IMRT, intensity modulated radiation therapy; VMAT, volumetric modulated arc therapy; Gy, Gray.

### 3.2 Machine characteristics

Table 7 shows average machine characteristic results for all machines related to geometry, kV‐CBCT system and treatment couch, including the variation between machines. Machine characteristic results of individual machines can be found in Table S8.

**TABLE 7:** Average [min–max] and standard deviation machine characterization results of all Ethos treatment machines. Section A shows results on geometric accuracy quantification, Section B shows results on kV‐CBCT system validation, and Section C shows results on treatment couch attenuation. Results of individual machines can be found in Table S8

A. Geometry		Average [min–max]	SD
Isocenter accuracy [mm]	Gantry rotation CW	0.31 [0.26–0.45]	0.07
	Gantry rotation CCW	0.29 [0.24–0.45]	0.08
	Collimator rotation G_0_	0.11 [0.09–0.17]	0.03
	Collimator rotation G_90_	0.13 [0.09–0.14]	0.02
kV‐MV isocenter coincidence [mm]	Lateral	−0.10 [−0.25–0.05]	0.12
	Longitudinal	−0.11 [−0.23–0.10]	0.14
	Vertical	0.30 [0.05–0.50]	0.15

B. kV‐CBCT system		**Average [min–max]**	**SD**
Beam energy [kV]	Mean deviation	1.06 [0.54–2.26]	0.63
Exposure time [ms]	Mean deviation	0.22 [0.09–0.80]	0.29
HVL [mm AL]	Mean deviation	0.49 [0.42–0.53]	0.04
CTDI_weighted_ [mGy]	Mean deviation	1.35 [0.85–1.57]	0.29

C. Treatment couch		**Average [min–max]**	**SD**
Attenuation [%]	Top: G_0_/G_180_	2.39 [1.80–2.94]	0.41
	Top: G_45_/G_225_	3.62 [2.76–4.35]	0.69
	Center: G_0_/G_180_	3.53 [2.72–4.33]	0.51
	Center: G_45_/G_225_	4.75 [3.85–5.25]	0.54

Abbreviations: SD, standard deviation; DLG, dosimetric leaf gap; mm, millimeter.

#### 3.2.1 Geometry

All machines show submillimeter isocenter accuracy for gantry and collimator rotations as well as kV‐MV coincidence (Table 7A). For gantry (collimator) rotations, an average isocenter accuracy of 0.30 mm (0.12 mm) was found. The largest kV‐MV isocenter discrepancy was found in vertical direction and resulted in a mean (maximum) isocenter coincidence of 0.30 mm (0.50 mm).

#### 3.2.2 kV‐CBCT system

Limited differences between the six kV‐CBCT systems were observed (Table 7B). The largest mean deviation between intended and measured energy peaks, exposure times and HVL was 2.26 kV, 0.80 ms and 0.53 mm AL, respectively. For all kV‐CBCT systems, Pearson correlation coefficients above 0.99 were found and indicated accurate exposure time linearity. The mean deviation between measured and vendor‐specified CTDI_weighted_ values ranges between 0.85 mGy and 1.57 mGy while the variation of 0.29 mGy showed an accurate agreement of CBCT imaging dose between machines.

#### 3.2.3 Treatment couch

For all machines, beam attenuation by the treatment couch increased for the center position compared to the top position (Table 7C). Beam attenuation for the perpendicular (oblique) beams ranges between 1.80%–2.94% (2.76%–4.35%) and 2.72%–4.33% (3.85%–5.25%) for the top respectively center position. The largest attenuation variation of 0.69% was obtained when using oblique beams at the top couch position.

## 4 DISCUSSION

In this first multi‐machine Ethos therapy system analysis, we evaluated the quality of six recently installed and accepted systems by characterizing essential machine components and quantifying variations between machines. Compared to vendor‐provided reference data, a distinct agreement in terms of dosimetric and mechanical characteristics was found for all machines. Furthermore, an adequate correspondence between dosimetric characteristics and the pre‐configured beam model was confirmed. Limited variations between treatment machines were observed in terms of dosimetric and mechanical performances, indicating good consistency among the six treatment machines. The high machine quality enables the application of online ART for treatment precision enhancement while machine similarity allows for patient interchangeability between treatment machines.

For all systems, our systematically obtained results were collected after successful machine acceptance. The extensive vendor acceptance testing does not cover all necessary aspects and therefore not sufficient prior to clinical introduction. Other vendor‐supplied tests (e.g., machine performance checks) provide useful information but is not completely independent and therefore not included in this study. Although all acceptance results were within the acceptance criteria as defined by the vendor, the relatively large acceptance criteria can still result in substantial deviations between machines. Nevertheless, our results show minimal variation between treatment machines for dosimetric and mechanical characteristics.

Beam characterization is performed by comparing open field dosimetry measurements of individual machines with the vendor‐provided reference data. All profiles of individual machines were obtained at the same time using the same type of equipment. Due to logistical reasons, either the PTW water tank and detector combination or the IBA water tank and detector combination was used. All profiles were collected in accordance with the vendor‐provided measurements to exclude a possible measurement bias. The type of detector used for crossline profiles measurements of small fields (≤5 × 5 cm^2^) was however not available in our department and the crossline profiles of small fields were therefore excluded from machine commissioning and consequently the analysis. Nevertheless, the substantial number of included profiles remained sufficient for adequate beam characterization of individual machines.

Several measurements were performed and compared to TPS‐based dose calculations for beam model verification, including point dose measurements. All point dose measurements were collected with a small ionization chamber in a water tank and compared to single point doses derived from dose calculations. Although calculated dose values should ideally represent the entire collecting volume of the detector, in this study point dose values were considered representative for the entire collecting volume of the ionization chamber. Since our measurements are performed in a homogeneous water medium using an ionization chamber with small collecting volume, differences in calculated dose for single points and small detector volumes are expected to be negligible. Furthermore, the Ethos TPS with the smallest available resolution of 2.5 mm limits the accuracy of calculated dose in small volumes. Note that the determined variation between treatment machines will not be influenced by TPS calculation results since only measurement results are compared.

The dual‐layer MLC system is characterized by determining the DLG and leaf transmission values. The DLG definition for a dual‐layer system is however challenging due to the interplay between leafs of both MLC banks. In this study, DLG values are therefore derived by duplicating the classical setup described for the single‐layer MLC systems using received DICOM files.^22^ The obtained DLG values for individual MLC layers and combined leaf banks are considered representative and also perfectly usable for machine comparison. The impact of trailing distance between leafs from different MLC layers was outside the scope of this study and therefore not derived for individual machines. Since similarity in leaf trailing behavior based on multiple machines is previously reported,^22^ the effect on DLG machine variation results is expected to be negligible. Leaf transmission characteristics are determined with a 26 × 26 cm^2^ field size as defined by the other MLC layer. Since MLC leaf transmission depends on the field size,^22^, ^26^ a worst‐case scenario is used by selecting a field size close to the maximum field size of the system. Nevertheless, DLG values as well as leaf transmission values found in this study are in line with previously reported values.^22^

The γ_1%/1mm_ profile results obtained for beam model verification indicated accurate agreements between calculated and measured dose profiles. All dose calculations were performed using the Ethos TPS with the smallest available resolution of 2.5 mm. Dose profiles were extracted from calculated 3D dose distributions and interpolated to a 1.0 mm resolution for gamma evaluation. For the 1 × 1 cm^2^ and 2 × 2 cm^2^ fields however the dosimetric accuracy is effected by the dose calculation resolution^13^ and this effect is also confirmed by our findings for small fields regarding the relatively large γ_1%/1mm_ results and substantial deviations from calculated D_10cm_ values. To determine machine variations for small fields without resolution limitations, additional γ_1%/1mm_ evaluations for corresponding measurements were performed and resulted in minor machine differences for small fields. Also, the standard deviation results for small fields (1 × 1 cm^2^, 2 × 2 cm^2^) indicated minimal machine variations.

Beam attenuation by the treatment couch is determined at two different couch positions because the thickness of the couch varies in longitudinal direction. The mean attenuation using perpendicular (oblique) beams is 2.38% (3.62%) at the top position and 3.53% (4.74%) at the central position. For treatment planning purposes, a vendor‐provided couch model is available in the TPS and contains a constant thickness in longitudinal direction. The calculated attenuation of 2.39% for perpendicular beams and 3.64% for oblique beams largely agrees with the measured attenuations at the top position. As a result, calculated beam attenuation is underestimated for treatments executed around the center position of the treatment couch.

For beam model validation purposes, calculated dose distributions of optimized treatment plans were compared to corresponding measurements for all machines and resulted in appropriate agreements in terms of γ_3%/3mm_ pass rates. Only one treatment plan (plan ID: 3) showed suboptimal results for the voxels in the high‐dose regions. Since treatment plans are optimized on patient geometry and recalculated and measured on the phantom geometry substantial deviations from the intended dose distribution including relocation of high‐doses can occur, especially for treatment plans with tangential fields. For the plan with suboptimal results, the phantom‐based dose distribution deviated from the intended dose distribution including relocation of the high‐dose region toward the phantom edge. Although this specific measurement could probably be improved by shifting the phantom to place the high‐dose region near the detector center, appropriate agreements will contribute to individual machine results while our overall plan results already showed consistency between machines.

Dose distributions measured on different machines were also used to verify the agreement in dynamic dose delivery between machines. Although all measurements were obtained according our clinical protocol (i.e., phantom positioning based on machine laser alignment without MV‐based position verification), phantom position errors became relevant in combination with our strict gamma criteria. To minimize the effect of submillimeter position errors, 3D alignment corrections were applied on a per phantom position basis resulting in adequate agreements between dose distributions in terms of γ_1%/1mm_ pass rates. Substantial machine variations (SD > 10) remained for both plans with the high‐dose region located towards the detector edge and mainly caused by results obtained at a specific machine. Therefore, it is likely that these deviations are largely related to the position of the measurement equipment related to the high‐dose region at this specific machine instead of the treatment machine.

## 5 CONCLUSION

This study demonstrates excellent agreement between individual Ethos therapy systems and vendor‐provided reference data as well as individual Ethos therapy systems and pre‐configured beam model in terms of dosimetric and mechanical characteristics. Furthermore, our results show good consistency among treatment machines and provide valuable insight on machine characteristics. The extensive and systematically obtained results provide benchmark data for future clinical introductions of Ethos therapy systems.

## AUTHOR CONTRIBUTION

All authors contributed to the conception of the work, acquisition, analysis, and interpretation of the data. Also, all authors contributed in writing the manuscript, approved the submitted version and agreed to be accountable for all aspects of the work.

## CONFLICT OF INTEREST

No conflicts of interest.

## Supporting information

Supporting Information
