# Supplementary material for: Characterization of Ethos therapy systems for adaptive radiation therapy: A multi‐machine comparison
Source: J Appl Clin Med Phys. 2023 Jan 17;24(5):e13905. doi: 10.1002/acm2.13905 (PMC10161062; doi:10.1002/acm2.13905)
Supplement: Supplementary file 1 — Supporting Information [file ACM2-24-e13905-s001.docx]

**Supporting Material**

**Characterization of Ethos therapy systems for adaptive radiation therapy:**

**a multi-machine comparison**

Agustinus J. van de Schoot^1^, Daan Hoffmans^2^, Karel M. van Ingen^1^, Martijn J. Simons^2^, Jan Wiersma^1^

*^1^Department of Radiation Oncology, Amsterdam University Medical Center – location University of Amsterdam, Meibergdreef 9, Amsterdam, the Netherlands*

*^2^Department of Radiation Oncology, Amsterdam University Medical Center – location Vrije Universiteit Amsterdam, Boelelaan 1117, Amsterdam, the Netherlands*

Journal of Applied Clinical Medical Physics, **Submitted 2022**.

**Supplementary material**

Supplementary Tables S1 – S8
